# Impact of Hormonal Replacement Therapy on Bone Mineral Density in Premature Ovarian Insufficiency Patients

**DOI:** 10.3390/jcm9123961

**Published:** 2020-12-07

**Authors:** Agnieszka Podfigurna, Marzena Maciejewska-Jeske, Malgorzata Nadolna, Paula Mikolajska-Ptas, Anna Szeliga, Przemyslaw Bilinski, Paulina Napierala, Blazej Meczekalski

**Affiliations:** 1 Department of Gynecological Endocrinology, Poznan University of Medical Sciences, 33 Polna Street, 60-535 Poznan, Poland; agnieszkapodfigurna@gmail.com (A.P.); marzena@jeske.pl (M.M.-J.); anna.mariaszeliga@gmail.com (A.S.); napierala.anna92@gmail.com (P.N.); 2 Students Scientific Society of the Department of Gynecological Endocrinology, Poznan University of Medical Sciences, 33 Polna Street, 60-535 Poznan, Poland; mnadolna@hotmail.com (M.N.); pola.wiktoria@wp.pl (P.M.-P.); 3 The President Stanislaw Wojciechowski State University of Applied Sciences in Kalisz, 62-800 Kalisz, Poland; bildom@gmail.com; 4 Copernicus Memorial Multidisciplinary Comprehensive Cancer and Traumatology Center, 93-513 Lodz, Poland

**Keywords:** premature ovarian insufficiency, DXA, osteoporosis, menopause, bone mineral density

## Abstract

Premature ovarian insufficiency (POI) is a type of hypergonadotropic hypogonadism caused by impaired ovarian function before the age of 40. Due to the hypoestrogenism, women with POI experience a variety of health complications, including an increased risk of bone mineral density loss and developing osteopenia and osteoporosis, which poses an important problem for public health. **Purpose:** The aim of this study was to evaluate and compare the values of bone mineral density (BMD), T-score and Z-score within the lumbar spine (L1-L4) using the dual energy X-ray absorptiometry method. The dual-energy X-ray absorptiometry (DXA) scans described in this original prospective article were performed at the time of POI diagnosis and after treatment with sequential hormone replacement therapy (HRT). **Materials and methods:** This study included 132 patients with a mean age of 31.86 ± 7.75 years who had been diagnosed with idiopathic POI. The control group consisted of 17 healthy women with regular menstrual cycles, with a mean age of 23.21 ± 5.86 years. Serum follicle-stimulating hormone (FSH), luteinizing hormone (LH), 17-estradiol (E2), prolactin (PRL), testosterone (T), dehydroepiandrosterone sulfate (DHEA-S), thyroid-stimulating hormone (TSH), free thyroxine (fT4), insulin, and fasting serum glucose were measured. Lumbar spine (L1-L4) BMD was assessed by means of dual-energy X-ray absorptiometry. DXA scans were performed at the time of diagnosis and following treatment with sequential hormone replacement therapy (HRT) comprised of daily oral 2 mg 17-β-estradiol and 10 mg dydrogesterone. The mean time of observation was 3 ± 2 years. **Results:** Patients in the POI group presented with characteristic hypergonadotropic hypogonadism. They had a significantly decreased mean lumbar spine BMD when compared to healthy controls (1.088 ± 0.14 g/cm^2^) vs. 1.150 ± 0.30 g/cm^2^) (*p* = 0.04) as well as a decreased T-score (0.75 ± 1.167 vs. −0.144 ± 0.82) (*p* = 003). There was a significant increase in BMD (1.088 ± 0.14 vs. 1.109 ± 0.14; *p* < 0.001), T-score (−0.75 ± 1.17 vs. −0.59 ± 1.22; *p* < 0.001), and Z-score (−0.75 ± 1.12 vs. −0.49 ± 1.11; *p* < 0.001) after the implementation of HRT when compared to pre-treatment results. **Conclusions:** In conclusion, this study has demonstrated that patients with POI often have decreased bone mineral density and that the implementation of HRT has a significant and positive influence on bone mass. The implementation of full-dose HRT and monitoring of bone status is particularly important in these patients.

## 1. Introduction

Premature ovarian insufficiency (POI) is a type of hypergonadotropic hypogonadism caused by impaired ovarian function before the age of 40 [1,2]. It affects 1:100 women before the age of 40 and 1:10,000 before the age of 20 [2]. Due to the hypoestrogenism, women with POI experience a variety of health complications. The natural progression of POI includes a short period of oligomenorrhea with subsequent secondary amenorrhea. If POI occurs before the age of 16 years, patients often present with primary amenorrhea. Vasomotor symptoms (hot flashes, night sweats), genitourinary symptoms (vaginal dryness, dyspareunia), neurological impairment, increased cardiovascular risk, and bone health deterioration are all part of the natural sequalae of POI [1]. 

The impact of estrogen deficiency on bone mass in postmenopausal patients is well described in the literature. Bone mass reduction progresses as a result of increased osteoclast action [3] causing cancellous bone perforation. Similarly, POI has adverse long-term effects on bone health. Various studies have demonstrated that patients with POI concomitantly develop decreased bone mineral density (BMD) [4]. Thus, according to recent discussions in the literature [5,6], it is reasonable to expect that these patients are likely to experience an increased risk of developing osteopenia and osteoporosis, and potential fractures in later life. 

Fracture prevention is a worldwide public health priority; osteoporosis is a major healthcare problem leading to a high incidence of various fractures causing morbidity and mortality in the ageing population. Therefore, care should be taken to provide patients with effective treatment to prevent long-term health complications. The efficacy of hormone replacement therapy (HRT) as a protection against adverse outcomes in POI, however, has been poorly evaluated. The potential benefits these patients can gain form HRT may not fully correspond with the body of trials carried out on populations of postmenopausal women [6].

HRT has a favorable effect on BMD in postmenopausal women and decreases their overall risk of bone fractures [7,8,9]. The evidence suggests that supplementing estrogen levels in POI and restoring it to normal levels through the use of HRT replaces ovarian function and may also have a positive impact on bone mass. Implementation of HRT raises the level of serum estradiol, which is a stimulus for bone formation and slows bone resorption [10]. 

Taking into consideration the limited data regarding BMD in POI and the influence of different HRT regimens on BMD in this group of patients, the aim of this study was to compare the BMD of POI patients with that of healthy controls and to evaluate the effect of sequential hormonal replacement therapy on BMD in this group of patients. The medical problem presented in this study represents the importance to public health of understanding the risks of osteopenia, osteoporosis in populations of young women with premature ovarian insufficiency (POI).

## 2. Materials and Methods

### 2.1. Materials

This study included 132 patients with a mean age of 31.86 ± 7.75 years. They had been diagnosed with spontaneous POI and treated by the Department of Gynecological Endocrinology, Poznan University of Medical Sciences, Poznan, Poland during the period from 2013 to 2016. BMD was assessed in each individual before and after the use of estro-progestin hormone therapy.

Qualifying participants had an established diagnosis of POI based on the following the criteria [11]

(1) Onset before 40 years of age
(2) Secondary amenorrhea for a duration of at least 4 months
(3) FSH level above 25 IU/L measured on two separate occasions at least 4 months apart
(4) Estrogen level below 50 pg/mL

To maintain homogeneity in the study population, only patients with an idiopathic etiology of POI were included. Subjects with karyotype abnormalities and genetic defects, and those with an autoimmune, infectious or iatrogenic etiology of POI were disqualified.

The control group consisted of 17 healthy women with a mean age of 23.21 ± 5.86 years and regular menstrual cycles.

Exclusion criteria for the control group were:

(1) Any preexisting endocrine disorders—based on laboratory parameters and anamnesis
(2) Chronic diseases, particularly diseases affecting the musculoskeletal system
(3) Any hormonal treatment taken in the 3 months preceding the examination.

### 2.2. Methods

Each qualifying study participant had a thorough medical history taken, and a physical and gynecological examination and a transvaginal ultrasound were performed. Weight (kg) and height (m) were measured and BMI was calculated (kg/m^2^). 

Venous blood samples were collected between 7:00 am and 9:00 am in a fasting state. Blood samples in the control group were drawn in the late follicular phase; between the 10th and 12th day of the menstrual cycle. 

Serum follicle-stimulating hormone (FSH), luteinizing hormone (LH), 17-estradiol (E2), prolactin (PRL), testosterone (T), dehydroepiandrosterone sulfate (DHEA-S), thyroid-stimulating hormone (TSH), free thyroxine (fT4), insulin, and fasting serum glucose were measured in all participants. Serum concentration was determined using electrochemiluminescence immunoassay (ECLIA) on a Cobas E601 analyzer (Roche Diagnostics, Indianapolis, IN, USA).

BMD measurement of the lumbar spine (L1-L4) was performed using dual-energy X-ray absorptiometry (DXA, Lunar Prodigy Primo, General Electric, USA). The results were expressed as g/cm^2^, Z-score, and T-score. The T-score was calculated from mean peak BMD and SD obtained from database analysis of normative data for the lumbar spine of young healthy adults. T-score values of between −1 and −2.5 were used as thresholds for diagnosing osteopenia, and values lower than −2.5 were characterized as osteoporosis. 

DXA was performed at the time of diagnosis and following treatment with sequential hormone replacement therapy (HRT). Treatment consisted of daily oral 2 mg 17-β-estradiol and 10 mg dydrogesterone. The mean time of observation was 3 ± 2 years.

This study may be limited by the inconsistent durations of hormone replacement therapy, as in some patients it was shorter, and in some it was longer; however, it lasted at least 12 months in all cases.

### 2.3. Statistical Analysis

Statistical analysis was performed using StatSoft 2012 STATISTICA Version 12. The normality of data distribution was assessed using the Shapiro–Wilk test. The Pearson’s linear correlation coefficient and Spearman’s rank correlation coefficient were used for correlation assessment. For comparison, Student’s t-test or the Mann–Whitney test were used where appropriate. Comparison between more than 2 groups was performed using analysis of variance (ANOVA test). A *p*-value of 0.05 was considered statistically significant.

All participants provided written informed consent before enrolling in the study. The study protocol was approved by the Ethics Committee of Poznan University of Medical Sciences in Poznan, Poland.

## 3. Results

Patients in the POI group presented with characteristic hypergonadotropic hypogonadism (significantly elevated serum concentrations of FSH and LH with concomitantly low serum level of estradiol). They were also found to have a significantly higher fasting glucose level when compared to healthy controls (Table 1). 

**Figure 1:**
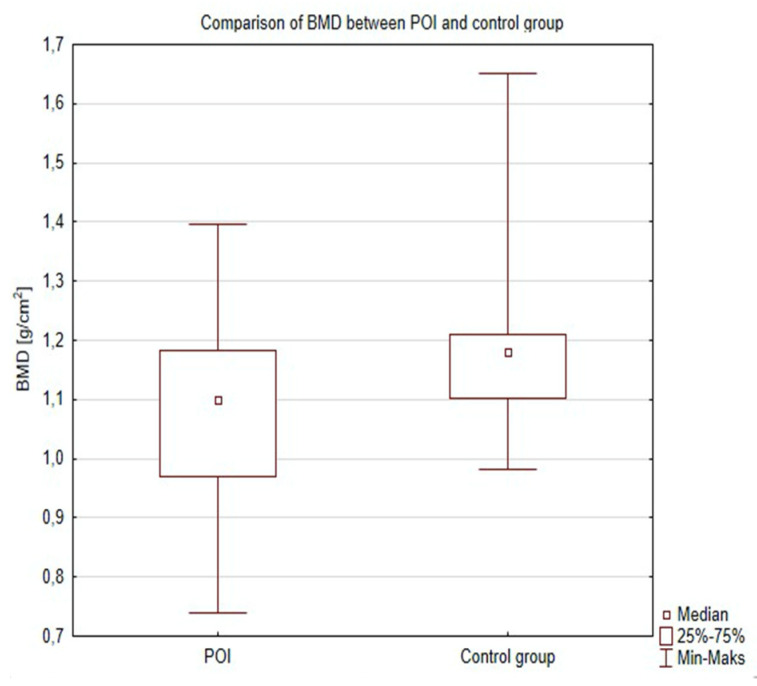
Comparison of BMD between POI and control group.

**Figure 2:**
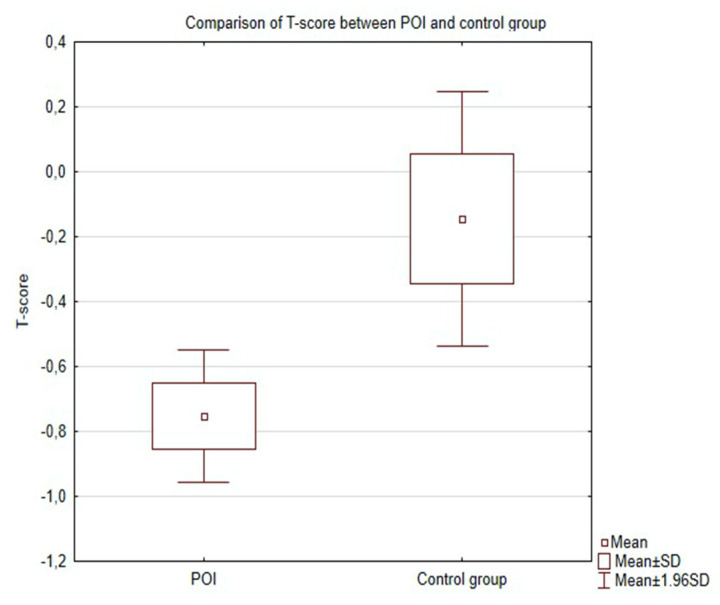
Comparison of T-score between POI and control group.

Women in the POI group had a significantly decreased lumbar spine mean BMD (1088 ± 0.14 g/cm^2^ vs. 1150 ± 0.30 g/cm^2^) (*p* = 0.04), (median 1099 g/cm^2^ vs. 1169 g/cm^2^ ) (Figure 1) and T-score (0.75 ± 1167 vs. −0.144 ± 0.82) (*p* = 0.03) when compared to healthy controls (Figure 2). 

Comparative analysis of BMD, T-scores, and Z-scores was performed in the study group. Values at baseline were compared with post-treatment scores and a significant increase in BMD, T-score, and Z-score was observed following implementation of HRT in these patients (Table 2).

Before commencing HRT, 8.94% of 132 patients in the POI group fulfilled the T-score criteria for osteoporosis and 30.08% were found to suffer from osteopenia. After HRT only 1.69% of the patients were found to meet diagnostic criteria for osteoporosis, whereas 42.37% were found to be osteopenic. 

The group of patients with POI was additionally divided into two groups based on baseline T-score values. The first subgroup included patients with a baseline T-score within the normal reference range. The second subgroup comprised POI patients with an abnormal baseline T-score defined as osteoporosis or osteopenia. It was noted, that the second subgroup (with abnormal baseline T-score) had a significantly higher serum LH concentration when compared to the group with normal baseline T-score. No additional differences were observed in other serum parameters. Moreover, analysis of variance found no significant difference between any measured parameter in the POI group when comparing normal BMD, osteopenia, and osteoporosis.

## 4. Discussion

POI is most often associated with impaired ovarian function and results in ovarian hormone deficiency. This lack of estrogens exerts an influence on osteoclasts, and leads to exacerbated bone reduction exceeding the rate of bone formation. As a consequence, estrogen deprivation in patients with POI has a negative impact on bone health, which may lead to decreased bone mineral density and an increased risk of osteoporosis and fractures in later life. 

This study was conducted to investigate the bone mineral density in a significant sample of patients with POI and compare it to age and weight-matched premenopausal controls. It then evaluated and quantified the influence of estroprogestative replacement therapy (using 17β-estradiol and dydrogesterone) on lumbar spine bone mass in this patient group.

Following this study, we report that women with POI have lower baseline lumbar spine BMD when compared to age and weight matched controls. The implementation of HRT increased BMD in these patients compared to their values at the baseline.

Soong et al. [12] was one of the first studies to evaluate BMD in premenopausal amenorrhoeic patients. They studied a group of 21 patients with premature ovarian insufficiency. The mean BMD for all participant groups in this study (including one group of patients with POI) was reduced when compared to the control group [12].

In another study, Popat et al. [13] compared a cohort of patients with spontaneous POI to age-matched women with regular menstrual cycles. They discovered that the POI population had a significantly lower (2–3%) BMD in the lumbar spine, femoral neck, and total hip. In total, 15% of women in the POI group were found to have a Z-score below −2.0, a result which was below the expected value for age. In total, 8% of women with POI were shown to have a T-score below −2.5, a result characteristic of osteoporosis [13]. 

A number of later studies went on to confirm the association between low BMD and POI patients: idiopathic POI [13], Turner syndrome [14], chemotherapy [15], ovariectomy [16], gonadal dysgenesis [17], and mixed etiology POI [18]. The results demonstrated in our study are reflective of the findings mentioned above and support the thesis that women with POI have a significantly decreased BMD and, in some cases, even T-score values in the osteopenic and osteoporotic range at the moment of diagnosis. These profound early changes may be explained by the fact that impairment of ovarian function and subsequent estrogen deficiency can proceed insidiously prior to the diagnosis of POI. Alzubaidi et al. [19] reports that diagnosis is delayed in up to half of cases, and in 25% of cases this delay can be more than 5 years. 

Popat et al. [13] have reported on certain modifiable factors correlating with significant low-for-age Z-score. These included vitamin D levels below 32 ng/mL, body weight below 55 kg, lack of regular exercise, calcium intake below 1000 mg/d, and no HRT implementation.

In order to protect bone health and decrease fracture risk, non-pharmacological strategies which are shown to be beneficial for postmenopausal women [20] are also advised for patients with POI. These recommendations include maintaining appropriate body weight, consuming a balanced diet, practicing weight-bearing exercises, moderating alcohol consumption, and smoking cessation [20]. According to WHO guidelines, maintaining a minimum recommended calcium intake of 1000 mg/day and a vitamin D intake of 800IU/day is essential to maintaining bone health and reducing the risk of fractures [21,22]. 

The results of BMD measurements should be taken into consideration at the time of management initiation for all patients, and particularly in those who present following a long period of estrogen deficiency and additional risk factors for decreased bone mass [23]. DXA is regarded as the gold standard in BMD assessment and is a first-line method for monitoring BMD in POI patients. It is important to note that the estimated yearly increase in BMD following the initiation of treatment is only 2%, which does not exceed the reported error margin of a DXA absorptiometer (1–2%). It is therefore recommended to perform a BMD assessment by DXA scan at 5 year intervals to ensure adequate monitoring [23,24]. The ideal measurement site for determining BMD has not yet been specified. It is known, however, that estrogens exert stronger antiresorptive action on trabecular bone, a structure making up a larger volume in the spine than in the total hip [25].

## 5. Estrogen Formulation

The use of estrogen therapy is an accepted method of improving POI-related health sequelae. This is in spite of the fact that trials comparing the efficacy of treatment regimens and their safety profiles with regard to symptom mitigation and the prevention of disease progression are deficient. Evidence has accumulated, however, showing that the use of HRT can restore bone mass in POI patients [26]. The available estrogen formulations commonly used to treat POI include 17β-estradiol (E2), ethinylestradiol (EE), and conjugated equine estrogens (CEE). A growing body of literature has supported the use of estradiol as the preferred form of estrogen replacement [11].

In 2014, Popat et al. [26] evaluated the influence of HRT on BMD in POI. In their study, preparations of 100 µg transdermal E2 and 10mg oral medroxyprogesterone were administered for a 3-year period. These were noted to significantly improve BMD measured at both the lumbar spine and femoral neck when compared to their baseline BMD values. The addition of testosterone did not have any beneficial effect on BMD levels in these patients. At the end of the study period, patients with POI exhibited no significant difference in BMD compared to the control group.

Similar results were observed by Cartwright et al. [27] who determined the superiority of HRT (2 mg oral E2 and 0.075 mg levonorgestrel daily) over a combined oral contraceptive pill (30 µg EE and 0.150 mg levonorgestrel daily for 21 days followed by a 7 day break) in improving the BMD of the lumbar spine in POI patients following 12 and 24 months of treatment. In POI patients receiving E2, significant gains in BMD were observed at all timepoints compared to EE. BMD in total hip and femoral neck did not differ significantly between the E2 and EE groups. The E2 group appeared to have a slightly greater influence on bone turnover markers (procollagen type I N-terminal propeptide (P1NP) and C-terminal telopeptide of type 1 collagen (CTX)) but statistical significance was not achieved. A possible explanation of these findings is the fact that EE leads to a considerable increase in the level of sex hormone binding globulin (SHBG) which decreases bioavailable estrogens and results in a reduced action on bone and BMD gain. 

Nevertheless, oral estrogen administration is subject to hepatic first-pass metabolism and has an inhibitory impact on insulin-like growth factor-1 (IGF-1) production, which is known to be an important factor in bone formation. Therefore, this has been proposed as the main reason oral use of estrogen in menopause suppresses IGF-1 levels, while estrogen taken through the transdermal route does not cause this downregulation. So far, it is still unclear whether this effect of the route-dependent metabolic action of estrogens can be extrapolated to patients with POI. Mauras et al. [28] analyzed metabolic rates and parameters in patients with Turner Syndrome who were undergoing growth-hormone treatment. They found no statistical difference in the level of the IGF-1 between patients receiving estrogen via oral and transdermal routes, despite higher serum estrogen levels after oral administration [28]. In a similar study, Nabhan et al. [29] noted no statistical difference in IGF-1 levels between patients with Turner Syndrome receiving estrogen by either route. Nevertheless, patients receiving estrogen transdermally were found to have significantly higher spine BMD compared to the group receiving estrogen via oral administration [29].

### Estrogen Dose

Current standards of practice dictate that the dose of exogenous estradiol should achieve replacement levels comparable to natural serum estrogen. Currently recommended formulations for patients with POI include daily doses of: 1–2 mg micronized oral E2; 100 µg transdermal E2; or 0.625–1.25 mg oral conjugated EE [1,30].

However, dose–response trials that include patients with POI are sparse and there is evidence supporting the observation that standard postmenopausal dosages are inadequate for patients with POI. In a study examining the etiology of idiopathic POI, Giraldo et al. [31] observed that standard HRT estrogen dosing (1 mg 17β-estradiol or 0.625 mg conjugated estrogen) was insufficient to sustain adequate BMD in POI patients. Moreover, the loss in BMD in these patients was similar or even more pronounced than that seen in women who did not receive estrogen therapy at all. 

Nevertheless, further studies are required to confirm this finding and extend its scope to patients with various other etiologies of POI. The results obtained in our study pertain to patients with idiopathic POI and provide support to observations that a 2 mg dose of 17β-estradiol is sufficient to restore bone mass in these patients.

## 6. Conclusions

In conclusion, this study demonstrates that patients with POI have decreased bone mineral density, and treatment with HT has the potential to greatly influence and improve bone mass. Further investigation, however, is needed to determine appropriate effective dosages.

## Figures and Tables

**Table 1:** Characterization of POI group and control group.

	POI Group			Control Group	
	Before Treatment	After Treatment	*p*1		*p*2
BMI [kg/m^2^]	23.68 ± 4.42	23.81 ± 4.67	0.25	20.71 ± 5.15	0.06
FSH [mIU/ml]	100.14 ± 36.93	82.56 ± 48.72	**<0.001**	9.83 ± 2.44	**<0.001**
LH [mIU/ml]	49.64 ± 18.02	50.67 ± 30.77	0.76	10.47 ± 2.62	**<0.001**
E2 [pg/mL]	13.43 ± 18.91	56.1 ± 68.83	**<0.001**	94.12 ± 24.86	**<0.001**
T [ng/mL]	0.28 ± 0.19	0.44 ± 1.09	**0.02**	0.32 ± 0.18	0.31
DHEA-S [µmol/L]	5.94 ± 2.89	5.98 ± 2.89	0.51	7.38 ± 4.79	0.46
fT4 [ng/dL]	1.29 ± 0.18	1.45 ± 1.19	0.24	1.23 ± 0.3	0.98
TSH [µIU/mL]	2.58 ± 1.94	2.1 ± 1.72	**0.01**	2.76 ± 1.69	0.53
Glucose [mg/dl]	90.45 ± 9.97	87.75 ± 14.43	0.35	71.32 ± 17.72	**<0.001**
Insulin [mU/mL]	8.9 ± 4.64	9.58 ± 6.9	0.28	7.43 ± 3.6	0.31
PRL [ng/mL]	12.35 ± 10.19	11.64 ± 6.07	0.82	8.42 ± 3.66	0.16

Data presented as mean ± SD; p1-comparison of data before and after treatment (comparisons made using the Wilcoxon signed-rank test); p2-comparison between TS before treatment and control group (comparisons made using t-student test (data consistent with normal distribution) or Mann-Whitney U test (data inconsistent with normal distribution)); *p* < 0.05 considered significant.

**Table 2:** BMD in POI group before and after treatment.

	POI Group		
	Before Treatment	After Treatment	*p*
BMD [g/cm2]	1.088 ± 0.14	1,109 ± 0.14	**<0.001**
T-score	−0.75 ± 1.17	−0.59 ± 1.22	**<0.001**
Z-score	−0.75 ± 1.12	−0.49 ± 1.11	**<0.001**

Data presented as mean ± SD; comparisons made using the Wilcoxon signed-rank test; *p* < 0.05 considered significant.
